# Endothelial cells exposed to atheroprotective flow secrete follistatin-like 1 protein which reduces transcytosis and inflammation

**DOI:** 10.1016/j.atherosclerosis.2021.08.025

**Published:** 2021-09

**Authors:** Mean Ghim, Kuin T. Pang, Sean A. Burnap, Ferheen Baig, Xiaoke Yin, Mehwish Arshad, Manuel Mayr, Peter D. Weinberg

**Affiliations:** a Department of Bioengineering, Imperial College London, London, UK; b King's College London British Heart Foundation Centre, School of Cardiovascular Medicine and Sciences, London, UK

**Keywords:** Wall shear stress, Permeability, Atherosclerosis, Inflammation, ICAM-1, VCAM-1, Transverse wall shear stress

## Abstract

**Background and aims:**

When endothelium is cultured in wells swirled on an orbital shaker, cells at the well centre experience putatively atherogenic flow whereas those near the edge experience putatively atheroprotective flow. Transcellular transport is decreased equally in both regions, consistent with it being reduced by a mediator released from cells in one part of the well and mixed in the swirling medium. Similar effects have been inferred for pro-inflammatory changes. Here we identify the mediator and flow characteristics stimulating its release.

**Methods and results:**

Medium conditioned by cells swirled at the edge, but not by cells swirled at the centre or cultured under static conditions, significantly reduced transendothelial transport of a low density lipoprotein (LDL)-sized tracer and tumor necrosis factor α (TNF-α)-induced activation and translocation of nuclear factor κB (NF-κB), adhesion molecule expression and monocyte adhesion. Inhibiting transcytosis similarly decreased tracer transport. Unbiased proteomics revealed that cells from the swirled edge secreted substantially more follistatin-like 1 (FSTL1) than cells from the swirled centre or from static wells. Exogenous FSTL1 reduced transport of the LDL-sized tracer and of LDL itself, as well as TNF-α-induced adhesion molecule expression. Bone morphogenetic protein 4 (BMP4) increased transport of the LDL-sized tracer and adhesion molecule expression; FSTL1 abolished these effects.

**Conclusions:**

Putatively atheroprotective flow stimulates secretion of FSTL1 by cultured endothelial cells. FSTL1 reduces transcellular transport of LDL-sized particles and of LDL itself, and inhibits endothelial activation. If this also occurs *in vivo*, it may account for the atheroprotective nature of such flow.

## Highlights

• Endothelial cells exposed to atheroprotective flow secrete FSTL1.
• FSTL1 reduces transendothelial transport of LDL and LDL-sized tracers.
• FSTL1 reduces the pro-inflammatory effects of TNF-α.

## Graphical abstract

**Figure:**
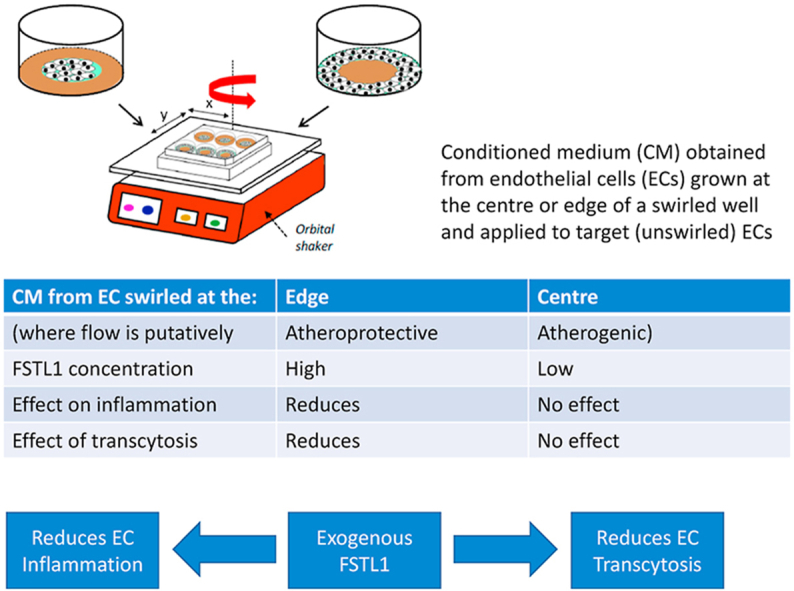

## 1 Introduction

Properties of endothelial cells (ECs) depend on the haemodynamic wall shear stress (WSS) to which the cells are exposed. Patterns of WSS vary from site to site within the arterial tree and the effect of this on local EC behaviour may explain the patchy distribution of atherosclerosis. However, the critical WSS characteristics have been a matter of debate over several decades. Low time average WSS [1] and/or highly oscillatory WSS [2] are widely assumed to trigger the disease. More recently, it has been shown that areas where WSS is multidirectional during the cardiac cycle have a particularly high prevalence of lesions [3].

Effects of atheroprotective and atherogenic flow characteristics can be studied using the “swirling well” method, in which ECs are grown in petri dishes or multi-well plates placed on the horizontal platform of an orbital shaker [4]. The wave induced by the swirling medium causes low magnitude, multidirectional shear towards the centre of the well, and high magnitude, uniaxial flow towards the edge. (The difference in magnitude and directionality between regions depends on the precise physical characteristics of the system [5].) Spatially resolved readouts of EC phenotype can be compared with the flow characteristics to which the cells have been exposed. Many studies have shown a decrease in homeostatic behaviour and an increase in potentially pathological behaviour from the edge to the centre of the well [reviewed in 4].

A potential problem with the method is that cells experiencing specific flow characteristics in one part of the well can release mediators that become well mixed in the medium and alter cell properties at other locations, confounding the true relation between WSS and EC behaviour. This can also be an advantage: by growing cells only in specific regions of the well, the effects of such mediators can be identified. We have demonstrated the existence of an anti-inflammatory mediator released into the medium by cells cultured at the edge of the well [6], and we have speculated that a secreted mediator similarly supresses transendothelial transport of LDL-sized particles [7].

Here we further extend the method by collecting medium conditioned by cells grown only in specific regions of the well and applying it to other ECs. In combination with unbiased proteomic techniques, this has enabled us to identify a hitherto unexpected molecule mediating barrier-tightening and anti-inflammatory effects of putatively atheroprotective flow.

## 2 Materials and methods

Methods are described in Supplementary Material.

## 3 Results

### 3.1 Region-specific cell culture

The choice of the “centre” and “edge” regions (radial distances <7.8 mm and >7.8 mm from the centre, respectively) was based on a computational fluid dynamics study that modelled swirling medium in a 12-well plate [8]. Cells cultured at the centre experienced more multidirectional shear than cells at the edge: the transverse WSS, a measure of multidirectionality [9], averaged 0.188 Pa in the centre region and 0.121 Pa in the edge region (55% higher in the centre). With the shaker and well characteristics used here, time average WSS showed a negligible difference between regions, averaging 0.268 Pa in the centre and 0.288 Pa at the edge (7% lower in the centre). The centre and edge regions had essentially identical areas of 191.3 mm^2^ and 192.3 mm^2^, respectively (0.5% difference).

Prolonged culture did not result in cell migration from the target area of the well into the passivated area (Supplementary Figure 2A and B).

### 3.2 Effect of conditioned medium on monolayer permeability

**Fig. 1:**
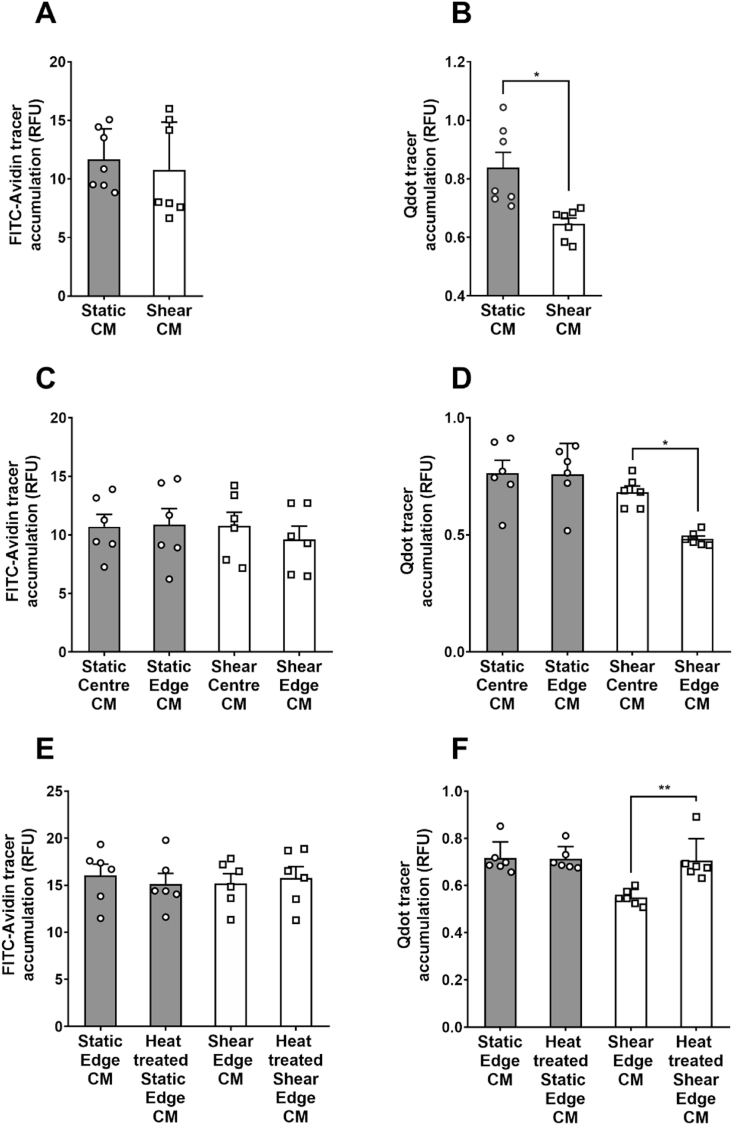
Accumulation of (A) FITC-avidin and (B) Qdot800-streptavidin tracers under PAEC monolayers cultured with medium conditioned by static or sheared PAEC monolayers. Effect of medium conditioned by sheared or non-sheared PAECs, cultured at the centre or edge of the wells, on accumulation of (C) FITC-avidin and (D) Qdot800-streptavidin tracers. Effect of heat-treatment on the influence of medium conditioned by PAECs grown under static conditions or sheared at the edge of the wells on accumulation of (E) FITC-avidin and (F) Qdot800-steptavidin tracers.

Transport of an albumin-sized tracer, fluorescein isothiocyanate-labelled avidin (FITC-avidin), across Porcine Aortic Endothelial Cell (PAEC) monolayers in static culture was not significantly reduced in medium conditioned by sheared PAEC monolayers, compared to medium conditioned by static PAEC monolayers (Fig. 1A). Transport of an LDL-sized tracer, Quantum Dot 800-labelled streptavidin (Qdot800-streptavidin), in contrast, was reduced 21.6 ± 4.7% (mean ± SEM, n = 7 isolations; *p <* 0.05) in medium conditioned by sheared PAEC monolayers, compared to medium conditioned by static PAEC monolayers (Fig. 1B). Images demonstrating that the paracellular route is used by FITC-avidin and the transcellular route by Qdot800-streptavdin transport are given in Ref. [7].

In subsequent experiments, the conditioned medium was obtained from cells cultured only at the centre or only at the edge of the well, either under static conditions or exposed to shear. There was no significant difference in FITC-avidin transport across monolayers treated with these four types of conditioned medium (Fig. 1C). Conversely, when transendothelial transport of Qdot800-streptavidin was examined, monolayers treated with medium conditioned by cells sheared at the edge of wells, and hence exposed to more uniaxial (i.e. putatively atheroprotective) flow, showed a 27.8 ± 3.1% decrease compared to monolayers cultured with medium conditioned by cells sheared at the centre of wells and hence exposed to multidirectional (i.e. putatively atherogenic) shear (Fig. 1D; n = 6 isolations, *p <* 0.05). There was no significant difference in Qdot800-streptavidin transport between monolayers treated with medium conditioned by cells sheared at the centre of swirled wells and by cells cultured under static conditions, either at the centre or the edge (Fig. 1D).

### 3.3 Effect of thermal denaturation on the barrier-tightening property of conditioned medium

Thermal denaturation of medium conditioned by cells sheared at the edge of the well did not alter its lack of effect on transendothelial transport of FITC-avidin (Fig. 1E) but abolished its ability to reduce transendothelial transport of Qdot800-streptavidin (Fig. 1F).

### 3.4 Effect of conditioned medium on monolayers treated with TNF-α

**Fig. 2:**
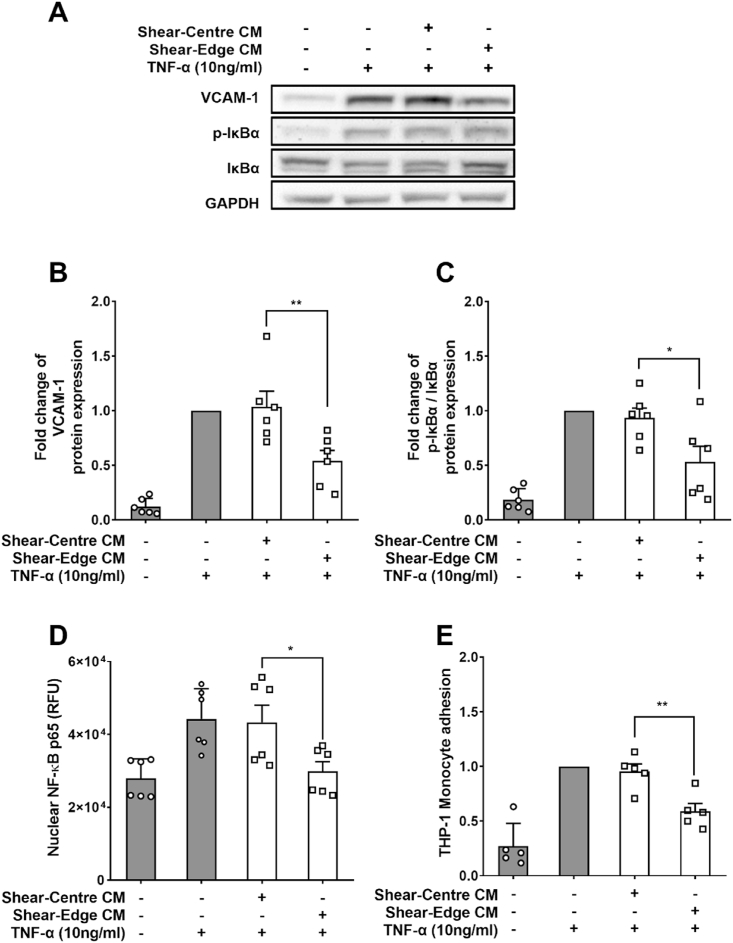
(A) Western blots of VCAM-1, p-IκBα, IκBα and glyceraldehyde 3-phosphate dehydrogenase (GAPDH). Western blot quantification for (B) VCAM-1 expression, (C) p-IκBα/IκBα ratio, (D) nuclear NF-κB p65, and (E) THP-1 monocyte adhesion, all for PAEC monolayers cultured in conditioned media from PAECs sheared in segmented wells, with and without added TNF-α.

TNF-α-treated PAEC monolayers cultured in medium conditioned by cells sheared at the edge of wells showed a 42.0 ± 11.3% decrease in vascular cell adhesion molecule 1 (VCAM-1) expression and a 41.6 ± 14.3% decrease in the ratio of phosphorylated to total NF-κB inhibitor α (p-IκBα/IκBα) compared to monolayers cultured in medium conditioned by cells sheared at the centre of wells (Fig. 2A–C; n = 6 isolations; *p <* 0.05, *p <* 0.01). Monolayers grown in medium conditioned by cells sheared at the edge of wells also had a 30.1 ± 4.2% decrease in nuclear NF-κB p65 (Fig. 2D; n = 6 isolations; *p <* 0.05) and a 37.1 ± 5.4% reduction in adhesion of the THP-1 monocytic cell line (Fig. 2E; n = 5 isolations; *p <* 0.01). We have previously shown that, in the absence of TNF-α, there is no significant effect of soluble mediators released in the swirling well on intercellular adhesion molecule 1 (ICAM-1) expression, VCAM-1 expression and monocyte adhesion [6].

### 3.5 Effect of size fractionation on the anti-inflammatory effect of conditioned medium

**Fig. 3:**
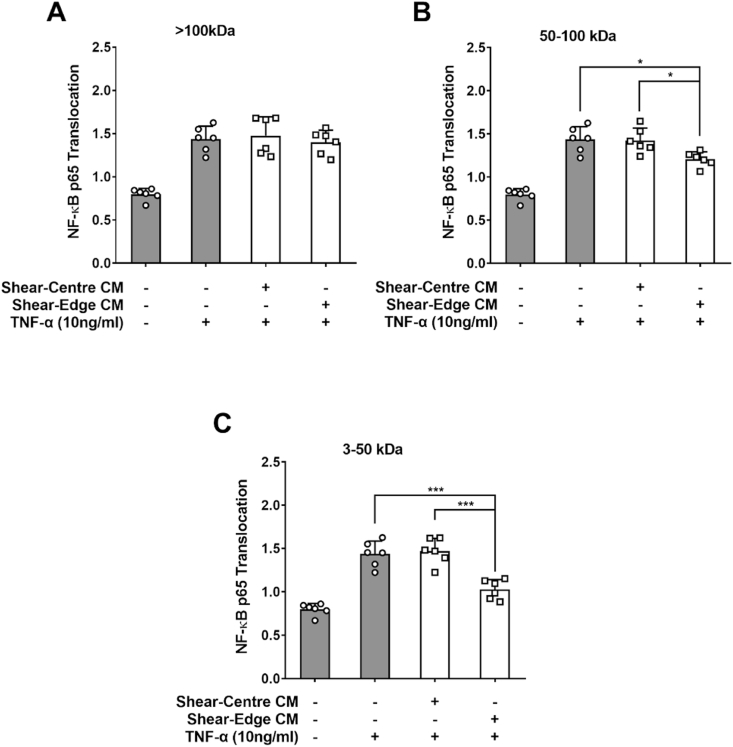
Effect of conditioned medium on NF-κB p65 nuclear translocation in PAECs treated with TNF-α. The medium was conditioned by PAECs sheared at the centre or the edge of the swirled well and then subject to sequential ultrafiltration so that it contained molecules (A) > 100 kDa, (B) 50–100 kDa and (C) 3–50 kDa.

There was no effect of a >100 kDa ultrafiltrate of medium condition by PAECs sheared either at the centre or at the edge of the well on TNF-α-induced NF-κB p65 nuclear translocation (Fig. 3A; n = 6 isolations; both *p* > 0.05).

A 50–100 kDa ultrafiltrate of medium conditioned by cells sheared at the edge of the well lowered translocation by 16.3 ± 3.4% compared to the translocation seen with the equivalent ultrafiltrate from cells sheared at the centre; similar reductions were seen compared to no ultrafiltrate, and there was no significant difference in translocation between the latter two conditions (Fig. 3B; n = 6 isolations; *p <* 0.05, *p <* 0.05 and *p* > 0.05 respectively).

3–50 kDa ultrafiltrates showed the same trend as the 50–100 kDa fractions but the effect size was doubled: ultrafiltrate from cells sheared at the edge lowered translocation by 31.3 ± 4.9% compared to ultrafiltrate from cells sheared at the centre; again, similar reductions were seen compared to no ultrafiltrate, and there was no difference between the latter two conditions (Fig. 3C; n = 6 isolations; *p <* 0.001, *p <* 0.001 and *p* > 0.05 respectively).

Since the cut-off of the filters is not precise [10], the fact that two of the fractions showed activity is consistent with the presence of one active component with a relative molecular mass around 50 kDa or with two or more active components with masses within the ranges 3–50 and 50–100 kDa.

### 3.6 Proteomic analysis of the secretome

Secretome analysis identified 65 proteins in medium conditioned from PAECs under the four conditions: sheared or static culture at the edge of the well, and sheared or static culture at the centre of the well – see Supplementary Table. One-way ANOVA showed statistically significant differences in normalised protein abundance between different conditioned media for three proteins: follistatin like-1 (FSTL1, *p =* 0.00057), basement membrane-specific heparan sulfate proteoglycan core protein isoform X4 (HSPG2, *p =* 0.019) and latent-transforming growth factor beta-binding protein 2 (LTBP2, *p =* 0.045).

**Fig. 4:**
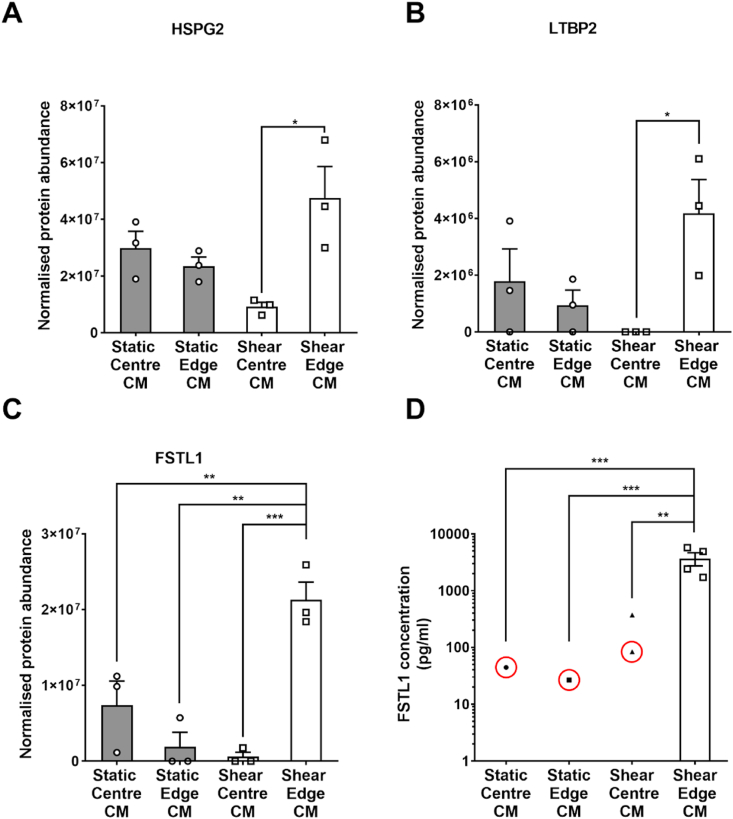
Normalised protein abundance of (A) HSPG2, (B) LTBP2 and (C) FSTL-1 detected by mass spectrometry in medium conditioned by PAECs grown at the centre or edge of static or sheared wells. (D) Concentrations of FSTL-1 determined by ELISA in medium conditioned by HAECs grown at the centre or edge of static or sheared wells. Red circles indicate values which were below the manufacturer's stated detection limit for the ELISA (312 pg/mL). Other samples from the static centre, static edge and sheared centre (n = 3, 3 and 2, respectively) gave no detectable signal and are not shown. (For interpretation of the references to colour in this figure legend, the reader is referred to the Web version of this article.)

Tukey's multiple comparison test was used to make further comparisons. The concentrations of HSPG2 and LTBP2 were significantly increased in medium from cells sheared at the edge compared to medium from cells sheared at the centre, but not compared to medium from static cells at the edge or centre (Fig. 4A and B; *p <* 0.05, *p >* 0.05 and *p >* 0.05, respectively, for both proteins). Only for FSTL1 was the concentration significantly increased in medium from cells sheared at the edge compared to medium from cells sheared at the centre and from static culture at both the edge and the centre (Fig. 4C; *p <* 0.001, *p <* 0.005 and *p <* 0.01, respectively). Furthermore, the relative molecular mass of HSPG2 is 470 kDa and that of LTBP2 is 191 kDa. Hence both would have been present in the >100 kDa fraction of conditioned medium, which was without effect. The mass of FSTL1 is 45–55 kDa, which lies at the border between the two smaller fractions.

### 3.7 Analysis by ELISA of FSTL1 concentration in conditioned medium

The concentration of FSTL1 in medium conditioned by cells sheared at the edge of the well averaged over 3000 pg/mL and all samples were above 1000 pg/mL. For the 12 samples obtained under the other three conditions, only one gave an FSTL1 concentration – 371 pg/mL – above the ELISA manufacturer's stated detection limit of 312 pg/mL. Three other samples gave values that were attributed concentrations (all below 100 pg/mL) by extrapolation of the calibration curve, and the remaining eight samples gave no measurable absorbance at all (Fig. 4D; n = 4; *p <* 0.01, *p <* 0.001 and *p <* 0.001 respectively).

### 3.8 Anti-inflammatory and permeability-reducing effects of FSTL1

**Fig. 5:**
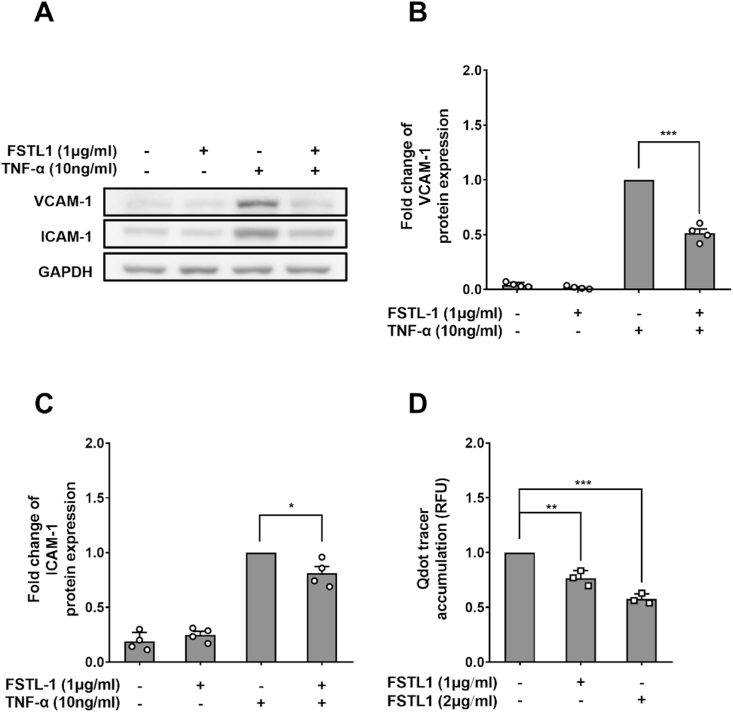
(A) Western blots of VCAM-1, ICAM-1 and GAPDH from HAECs treated or not treated with FSTL1 and TNF-α. Quantification of western blots for (B) VCAM-1 and (C) ICAM-1 in HAECs. (D) Qdot800-streptavidin tracer accumulation under HAEC monolayers after treatment with 1 or 2 μg/mL FSTL-1, normalised by accumulation after no addition.

VCAM-1 expression by Human Aortic Endothelial Cells (HAECs) preconditioned with human FSTL1 for 24 h followed by 24 h of TNF-α treatment, still with FSTL1 present, was reduced 48.7 ± 3.9% compared to HAECs treated only with TNF-α (Fig. 5A and B; n = 4; *p <* 0.001). A smaller, 19.1 ± 6.1% but still significant effect was seen for ICAM-1 (Fig. 5A and C; n = 4; *p <* 0.05). FSTL1 reduced transendothelial transport of Qdot800-streptavidin in a dose-dependent manner, by 23.2 ± 4.2% at 1 μg/mL and by 41.3 ± 2.7% at 2 μg/mL, respectively, compared to HAEC monolayers not treated with FSTL1 (Fig. 5D; n = 3; *p <* 0.01 and *p <* 0.001 versus control, respectively).

### 3.9 Effect of FSTL1 on transendothelial transport of LDL

**Fig. 6:**
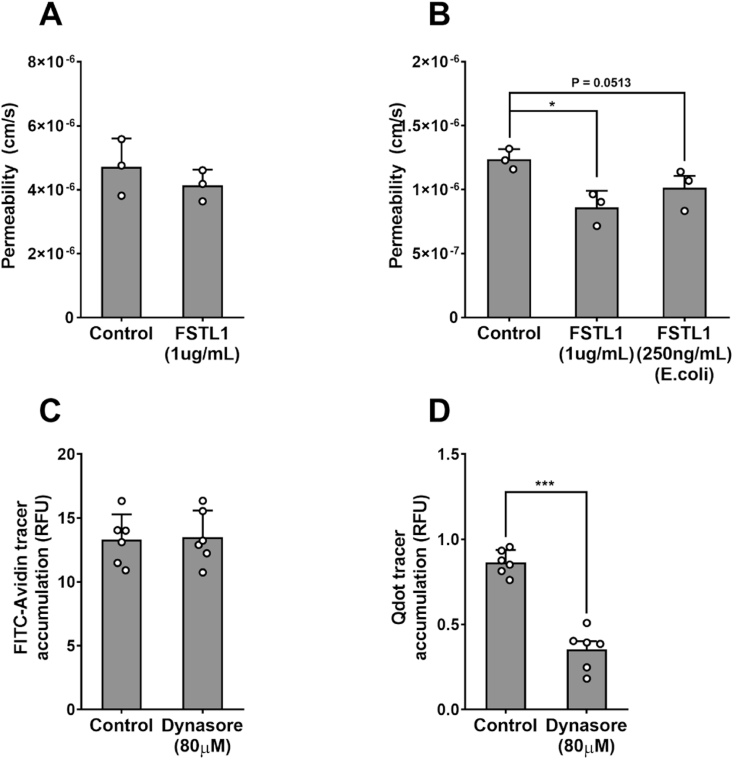
(A) Effect of 1 μg/mL FSTL1 on permeability of HAEC monolayers to FITC-Avidin. (B) Effect of 1 μg/mL FSTL1 or 250 ng/mL non-glycosylated FSTL1 on permeability of HAEC monolayers to LDL. Effect of 80 μM Dynasore on accumulation of (C) FITC-Avidin and (D) Qdot 800-streptavidin under PAEC monolayers.

1 μg/mL of glycosylated FSTL1 treatment did not reduce FITC-avidin transport across HAEC monolayers, but it reduced LDL transport by 31.28 ± 5.92% (Fig. 6A and B; n = 3; *p >* 0.05 and *p <* 0.05). Monolayers treated with non-glycosylated FSTL1 at the same concentration showed cell detachment and death, suggesting that the non-native form of the protein is cytotoxic. Treatment with the lower concentration of 250 ng/mL avoided this problem and there was a trend towards reduced LDL transport (by 17.24 ± 4.83%; Fig. 6B; n = 3; *p =* 0.0513).

### 3.10 Effect of Dynasore on transport across endothelium

Treatment of PAECs with Dynasore reduced Qdot800-streptavidin transport by 58.74 ± 6.33% compared to untreated PAEC monolayers but had no effect on FITC-avidin transport. (Fig. 6C and D; n = 6; *p <* 0.001 and *p >* 0.05).

### 3.11 BMP4 expression and secretion

BMP4 expression was detected in cells cultured under static conditions. Expression was not significantly altered in cells sheared at the centre of the well but was halved in cells sheared at the edge (Supplementary Figure 4A and B; *p <* 0.01 for edge *vs* centre; *p <* 0.05 for edge *vs* static). BMP4 concentrations were similarly lower in medium conditioned by cells sheared at the edge of the well than in medium conditioned under static conditions (*p <* 0.05), but the highest concentrations were seen in medium conditioned by cells sheared at the centre of the well (Supplementary Figure 4C; *p <* 0.05 *vs* static; *p <* 0.1 *vs* edge).

### 3.12 Effects of BMP4 and their inhibition by FSTL1

**Fig. 7:**
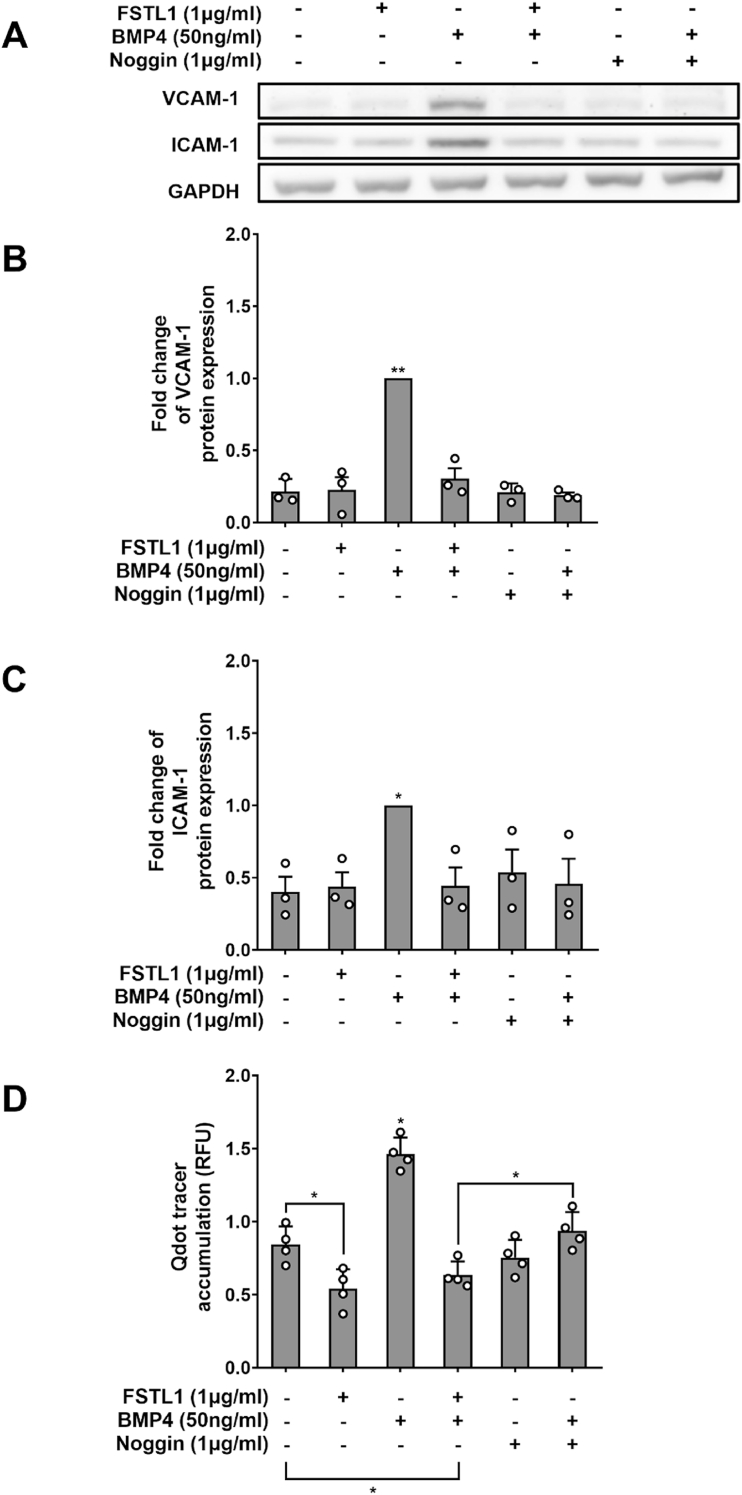
(A) Western blots of VCAM-1, ICAM-1 and GAPDH in HAECs exposed to BMP4, noggin and/or FSTL1. Effect of BMP4, Noggin and/or FSTL1 on expression of (B) VCAM-1 and (C) ICAM-1 in HAECs, quantified from western blots, and on (D) accumulation of Qdot800-streptavdin under HAEC monolayers.

BMP4 (50 ng/mL) significantly increased expression of VCAM-1 (Fig. 7A and B; n = 3; *p <* 0.05) and ICAM-1 (Fig. 7A and C; n = 3; *p <* 0.01). FSTL1 or the BMP inhibitor noggin at 1 μg/mL returned this elevated expression to baseline; neither had any effect on baseline expression (Fig. 7B and C).

BMP4 (50 ng/mL) substantially elevated transport of Qdot800-streptavidin (Fig. 7D; n = 4; *p <* 0.05). Noggin at 1 μg/mL abrogated this effect. FSTL1 at the same dose not only abrogated the effect but reduced transcytosis below baseline levels (*p <* 0.05), as also seen in Fig. 5, Fig. 6B, and below the level seen with noggin (Fig. 7D; *p <* 0.05).

## 4 Discussion

In a previous paper [7], we showed that when fluid dynamic shear stress was applied to cultured endothelial monolayers by swirling them on an orbital shaker, their permeability to LDL-sized particles decreased compared to the permeability seen in static culture. Permeability was reduced uniformly despite the markedly different shear stress profiles at the centre and edge of the well. That led us to speculate that a mediator was released by cells experiencing one type of shear profile, that it was well mixed in the swirling medium and that it decreased permeability by the same amount at all locations. The postulated mediator appeared specifically to affect transport of large particles, since the transport of an albumin-sized tracer did show regional differences, being higher in the centre of swirled wells and lower at the edge compared to static culture [7].

We subsequently developed methods that allow cells to be grown only at the edge or only at the centre of the wells, even under prolonged culture, and used these methods to investigate further the concept of a mediator secreted in a shear-dependent manner by examining effects of shear on inflammatory markers [6]. There was no difference in TNF-α-induced ICAM-1 or VCAM-1 expression between the centre and the edge when the whole well was seeded with cells and swirled. When only the centre or only the edge was seeded, expression was unchanged at the edge but elevated in the centre. This is consistent with an anti-inflammatory mediator being released from cells at the edge. When the whole well is seeded, it would limit expression of adhesion molecules at both the centre and the edge of the well. Only in the absence of this crosstalk would the cells exposed to the putatively atherogenic flow in the well centre have significantly higher adhesion molecule expression than the cells exposed to putatively atheroprotective flow at the well edge.

The present study further investigated the role of secreted mediators in regulating permeability to LDL-sized particles and LDL itself, and in regulating pro-inflammatory changes, by treating endothelium with medium conditioned by cells sheared on the orbital shaker; the cells were seeded either across the whole well or only at the edge or centre. Medium from static culture was used as a control. Consistent with our earlier study [7], we found that conditioned medium from fully seeded, swirled wells decreased permeability to LDL-sized particles in static culture; permeability to a smaller tracer was unaffected. We further showed that this property resulted from a mediator released by cells at the edge of the well, not by those at the centre. Parallel decreases were obtained for TNF-α-induced expression of VCAM-1, phosphorylation of IκBα, nuclear concentration of NF-κB p65 and adhesion of THP-1 monocytes.

Heat treatment, size fractionation and unbiased analysis of conditioned medium by mass spectrometry identified FSTL1, a secreted glycoprotein, as a candidate mediator; of the three proteins that differed in concentration between medium collected under different conditions, only FSTL1 was significantly increased in medium from cells exposed to putatively atheroprotective shear compared to all other treatment groups. ELISA of conditioned medium confirmed this result. Furthermore, the other two proteins were much larger and hence would have been present in the > 100 kDa fraction that was found not to alter an inflammatory marker.

In the absence of commercially available neutralising antibodies to FSTL1, we adopted the strategy of adding exogenous FSTL1 to cells in static culture. FSTL1 reduced TNF-α-induced VCAM-1 and ICAM-1 expression. These data plausibly explain our previous work [6] on the modulation of pro-inflammatory changes by shear stress *in vitro*. Other pathways may also be involved. Moreover, FSTL-1 acts on the NLR family pyrin domain containing 3 (NLRP3) inflammasome to promote IL-1β secretion from monocytes/macrophages [11] so it may have pro- as well as anti-inflammatory effects.

Exogenous FSTL1 also reduced endothelial permeability to the LDL-sized particle, Qdot800-streptavidin. We interpret this as a reduction in transcellular transport. The smaller FITC-avidin tracer is transported via junctions between endothelial cells [7,12] and was used to control for decreased paracellular permeability. It was unaffected by any conditioned medium or by exogenous FSTL1. The distribution of Qdot800-streptavidin observed in our earlier study [7] is consistent with its transport by a transcytotic mechanism: accumulation occurred under ECs and was reduced under their nuclei; it was not enhanced under cell junctions even after prolonged incubation. Additional evidence from the present study is that Dynasore substantially reduced subendothelial accumulation of the tracer, but not of FITC-avidin. Dynasore is an inhibitor of the GTPase domain of dynamin and hence of the fission of clathrin-coated vesicles from the plasma membrane [13]; it also causes dynamin-independent inhibition of fluid-phase endocytosis and micropinocytosis [14,15]. The existence of receptor-mediated transcytosis of Qdot800-streptavidin is improbable, so fluid-phase transcytosis is the most likely route.

We expect LDL to be transported by the same pathway. Early evidence for this derived from electron microscope studies [16]. The major transporter appeared to be uncoated plasmalemmal vesicles (broadly equivalent to caveolae in endothelial cells [17]); the process did not involve high-affinity binding, was not saturable and was unaffected by cooling to 4 °C. More recently, the role of receptors in LDL transcytosis has come to the fore, with evidence for a dependence on the LDL receptor in endothelium of the blood brain barrier [18], and on scavenger receptor class B type 1 (SR-B1) [[19], [20], [21]] and activin receptor-like kinase 1 (ALK1) [22] in coronary artery and aortic endothelia. These studies have tended to use sub-physiological LDL concentrations or cold washes that may have led to an underestimation of the role of receptor-independent, fluid-phase endocytosis. Even under these conditions, a 50-fold excess of unlabelled LDL, which will have abrogated any association with rare high-affinity receptors, left 30% of transcytotic transport intact [19]. Hence it is plausible that fluid-phase transcytosis plays a substantial role [23].

We studied LDL transport without labelling the particle, to avoid changes in charge or conformation that might alter its binding to scavenger or other receptors. The LDL was used at human physiological concentration; its transport across ECs cultured on Transwell filters was assessed by ELISA of medium in the sub-endothelial compartment 1 h after adding it to the medium above the cells. As with the Qdot800-streptavidin tracer, transendothelial transport was significantly reduced by FSTL1, and to approximately the same extent. Note that the Qdot800-streptavidin and LDL concentrations we measured do not include accumulation within the cells.

Since other effects of FSTL1 are dependent on its glycosylation state [reviewed in 24], we also investigated the effect on LDL transport of FSTL1 obtained by expression in a non-mammalian system. FSTL1 produced in *E. coli* was poorly tolerated by the human endothelial cells and only 25% of the dose of the mammalian FSTL1 could be used. Nevertheless, a reduction in LDL transport of borderline significance was obtained.

We conducted a preliminary investigation of signalling events downstream of FSTL1. We examined the role of BMP4 because this protein is known to be pro-inflammatory, to have higher expression in atheroprone than protected regions of the arterial tree, to have higher expression in the centre than the edge of swirling wells, and to be inhibited by FSTL1 [[25], [26], [27]]. We confirmed that intracellular BMP4 levels are higher in the centre than at the edge of the swirling well and we showed that the concentration of BMP4 in medium also depends on applied shear stress: compared to static culture, concentrations were higher in medium conditioned by cells sheared at the centre of the well and lower if the cells were sheared at the edge. We also verified that exogenous recombinant human BMP4 increases expression of adhesion molecules, and we additionally showed that it increases transport of Qdot800-streptavidin. The concentrations in medium of endogenous BMP4 were low (less than 5% of the maximum FSTL1 concentration) and the differences between conditions were smaller than those seen for FSTL1. This presumably explains why BMP4 was not detected by the proteomic analysis and why concentrations of BMP4 in the various conditioned media do not account for the differences in Qdot800-streptavidin transport.

FSTL1 blocked both the pro-inflammatory and the barrier-disrupting effects of exogenous BMP4, as did the BMP inhibitor noggin. An intriguing result was that whereas FSTL1 reduced transport of the LDL-sized tracer below baseline levels, noggin did not. Thus, in addition to blocking effects of BMP4, there appears to be at least one other pathway by which FSTL1 affects permeability. Note that noggin does not inhibit BMP9, and that FSTL1 binds to Alk1 [28]; both BMP9 and ALK1 are involved in the regulation of transcytosis [22,29].

Effects of shear on *FSTL1* expression have been investigated in previous *in vitro* studies. Holliday et al. [30] scraped endothelial cells from the human aortic valve and exposed them *in vitro* to either unidirectional shear (20 dyne/cm^2^) or oscillatory shear (±5 dyne/cm^2^, 1 Hz). There was no effect on *FSTL1* mRNA levels, regardless of the side of the valve from which the cells came. White et al. [31] exposed HUVEC to unidirectional shear of 15 or 75 dyne/cm^2^ and examined mRNA with a microarray that contains an assay for the *FSTL1* gene. No difference in expression was detected. Neither study included a static control and their negative results are consistent with our view that more complex aspects of flow such as its directionality are important. One possible upstream mediator is transforming growth factor β (TGF-β), which increases FSTL1 expression in numerous cell types and in ECs is produced in response to shear, an effect that is dependent on potassium channels [32]. We additionally note that disruption of the primary cilium, a known transducer of haemodynamic WSS, reduces *FSTL1* mRNA in an epithelial cell line [33].

We speculate that endothelial FSTL1 secretion also depends on WSS characteristics *in vivo*, with more being released in regions of uniaxial WSS than in regions of multidirectional WSS. Evidence supporting this view was obtained in the study of aortic valve ECs by Holliday et al. [30]: *FSTL1* mRNA levels were significantly lower in cells isolated from the aortic side than the ventricular side. The cells isolated from the aortic side had been exposed *in vitro* to oscillatory flow along one axis while the cells isolated from the ventricular side had been exposed to steady flow along one axis but, as explained above, this did not lead to the differential expression. The difference can therefore be attributed to the origin of the cells. The disease-resistant ventricular surface of the valve is exposed to predominantly uniaxial flow, whereas the disease-prone aortic surface is exposed to multidirectional flow [34]. Thus, consistent with our study, the cells exposed to multidirectional flow *in vivo* had reduced *FSTL1* expression.

A corollary of this speculation is that FSTL1 production may help explain WSS-related variation in macromolecule uptake by the arterial wall, variation in the propensity for pro-inflammatory changes, and variation in the prevalence of atherosclerotic lesions. A further inference is that FSTL1 itself or a compound acting in the same way could have a therapeutic role, reducing levels of disease in atheroprone regions of the arterial tree towards those seen in protected regions.

### 4.1 Limitations

Conditioned medium was assayed only for protein mediators. Other possible mediators include lipids, microparticles and microRNAs. (*FSTL1* mRNA can produce FSTL1 or miR-198, depending on the cellular environment [35], so a reciprocal relationship of FSTL1 and miR-198 concentrations in conditioned medium is possible.)

The maximum concentration of FSTL1 measured in conditioned medium was ≈6 ng/mL whereas the concentration of exogenous FSTL1 that produced a similar reduction in Qdot800-streptavdin transport was 1 μg/mL; 50 ng/mL was ineffective (data not shown). There were numerous differences between experiments applying conditioned medium and exogenous FSTL1, including that conditioned medium was applied for 6 days whilst exogenous FSTL1 was applied for 24 h, but the discrepancy could also have resulted from the use of a recombinant protein. Such proteins can have incomplete folding or aberrant glycosylation and may therefore be less active. The protein used in the present work has been used at similar doses in other studies [36,37] but further investigation of this topic is required. For example, a protein mediator at low concentration or a non-protein mediator may have been missed.

Data in the present study were generated using an *in vitro* model. Earlier data are consistent with the *in vivo* relevance of our findings but further investigation *in vivo* is required.

## Financial support

This study was supported by BHF Project Grant PG/15/102/31890.

## Author contributions

MG and PDW conceived the work and designed the experiments. MG conducted the majority of the study, assisted by KTP. MA carried out the computational fluid dynamics. SAB, FB and XY, supervised by MM, designed and conducted the proteomics analysis.

## Declaration of competing interest

The authors declare that they have no known competing financial interests or personal relationships that could have appeared to influence the work reported in this paper.
