# Supplementary material for: Endothelial cells exposed to atheroprotective flow secrete follistatin-like 1 protein which reduces transcytosis and inflammation
Source: Atherosclerosis. 2021 Sep;333:56–66. doi: 10.1016/j.atherosclerosis.2021.08.025 (PMC8459397; doi:10.1016/j.atherosclerosis.2021.08.025)
Supplement: Multimedia component 1 [file mmc1.docx]

**SUPPLEMENTARY MATERIAL**

**Methods**

***Cell culture***

Porcine aortic endothelial cells (PAECs) were isolated and their purity assessed as previously described [1]. They were cultured in Dulbecco's Modified Eagle Medium (DMEM) supplemented with 10% fetal bovine serum (FBS), 2.5 μg/mL amphotericin B, 100 U/mL penicillin, 100 μg/mL streptomycin, 50 μg/mL gentamycin, 5 mM L-glutamine, 90 μg/mL heparin and 5 μg/mL endothelial cell growth factor at 37 °C under 5% CO_2_. Medium was replaced every 2-3 days until the cells were confluent. Once confluent, cells were seeded in 12-well plates, which were either fully coated with 1% gelatin or partially coated at the centre or at the periphery of wells and passivated elsewhere as described in the next section. PAECs of passage 2 were used for experiments.

Human aortic endothelial cells (HAECs, PromoCell) were cultured in Endothelial Cell Growth Medium MV (ECGM, PromoCell). All experiments were carried out using HAECs of passage 5.

Human cells were used in experiments where human LDL or recombinant human FSTL1 or BMP4 were employed. PAECs were used in other experiments.

***Region-specific coating of 12-well culture plates***

To grow cells only at the centre or only at the edge of the well, even during prolonged culture, regions where cell growth was not wanted were passivated with a surface coating. Spreading of cells was also prevented by using plasticware that had not been cell-culture treated.

A mixture of polydimethylsiloxane (PDMS) base and curing agent (10:1 w/w) was mixed with iron (II,III) oxide powder (Inoxia) at 1:5 w/w and the final mixture poured into a 3-D printed mould, degassed and cured at 80°C for 2 h. CAD diagrams of the mould and the masks it produces are shown in Supplementary Figure 1. The mould was designed in PTC Creo and printed using an Ultimaker 2+ 3-D printer with polylactic acid filaments.

The PDMS masks were removed from the moulds, placed in 12-well plates and secured with two N42 neodymium magnets (12 mm diameter, 2 mm thickness) placed underneath the wells. The exposed surface of the wells was coated with 1% gelatin. The gelatin solution and masks were then removed, the wells washed with PBS three times and the uncoated surface of the wells passivated with 1% pluronic-F127 for 1 h at room temperature. The wells were again washed with phosphate buffered saline (PBS) three times and either stored with PBS in the wells at 4 °C or seeded with cells immediately.

Further details are given elsewhere [2, 3]. Reference [2] shows not only that there was no spread of the cells but also that there were no significant effects of soluble mediators on cell number, orientation and shape index.

***Application of shear stress***

12-well plates containing confluent monolayers and medium with an average depth of 2 mm were placed on the horizontal platform of an orbital shaker (PSU-10i, Grant Instruments). The platform translated with a circular orbit of diameter 10 mm at a rotational rate of 150 rpm.

***Collection, preparation and use of conditioned medium***

For each set of experiments, PAECs were cultured until confluent in two 12-well plates coated with 1% gelatin (donor plates) and in a third plate coated with biotinylated gelatin for tracer transport studies or normal gelatin for inflammation studies (target plate). For one of the two donor plates, shearing commenced straight away, and for the other it was delayed by 24 h. After 72 h of shearing, medium was replaced and the cells sheared for a further 48 h. The resulting conditioned medium was collected. Fresh medium was added to the wells, the shearing of the donor plates was continued and more conditioned medium was collected after a further 48 h. Donor plates cultured under static conditions provided control conditioned medium. In all cases, the conditioned medium was centrifuged (210 x *g*) to remove non-adhered cells and the supernatant was applied to the target plate, which was not sheared. The 24 h lag between the two donor plates meant that fresh conditioned medium could be applied to the target plate daily for 4 days.

*For tracer transport studies:*

For the 5^th^ day of conditioned medium treatment, target plates were cultured in conditioned medium produced by culturing donor plates in DMEM supplemented with 5% instead of 10% FBS. Tracer experiments were carried out after 24 h.

*For inflammation studies:*

For the 5^th^ day, target plates were cultured in conditioned medium with added TNF-α (10 ng/mL) for 24 h.

*For thermal denaturation of proteins in conditioned medium:*

Conditioned medium was heated for 30 minutes at 90°C, cooled and mixed with fresh medium before application. The final solution comprised 70% heat-treated and 30% fresh medium.

*For fractionation of proteins in conditioned medium:*

Conditioned medium was sequentially passed through 100, 50 and 3 kDa cut-off Amicon filters (Sigma-Aldrich). The concentrate from each stage was collected, diluted back to the original starting volume in fresh medium and applied to the experimental plates. TNF-α (10 ng/mL) was added to the target plates on the 5^th^ day of conditioned medium treatment.

***Tracer accumulation studies***

In our methods [4], derived from those of Dubrovskyi et al. [5], fluorescent tracers based on avidin are added to the medium above ECs grown on a biotinylated substrate. When the tracer crosses the endothelium, it binds to the substrate; after washing to remove any tracer remaining in the medium or inside cells, the fluorescence can be quantified. The size of the tracer is modified by altering the size of the fluorescent label. Small labels such as fluorescein give tracers having the size of albumin, whereas large labels such as quantum dots give LDL-sized tracers [4].

24 h before tracer studies, monolayers were cultured in reduced-serum media (5% FBS for DMEM and 2.5% for ECGM), after which 0.38 μM fluorescein isothiocyanate (FITC)-labelled avidin or 0.076 μM Quantum Dot 800 (Qdot800)-labelled streptavidin in DMEM or ECGM was applied for 3 minutes to monolayers cultured on biotinylated gelatin. Tracer solution was then removed, and the monolayers were rinsed with PBS three times and fixed with 4% paraformaldehyde. Tracer that had crossed the monolayer and accumulated underneath it was measured using a scanning fluorimetric plate reader (SpectraMax M5).

FITC-avidin and Qdot800-streptavidin differed not only in the size of the label but also in the protein moiety: avidin is a glycoprotein, whereas the bacterial streptavidin is not. However, we have previously compared avidin with NeutrAvidin, another deglycosylated form, when both were labelled with FITC, and found only minor differences in their pattern of transport [4].

***Detection of adhesion molecules and Iκbα phosphorylation***

Cells were lysed using radioimmunoprecipitation assay buffer (Sigma-Aldrich) supplemented with Halt protease and phosphatase inhibitor (ThermoFisher). Extracted proteins were separated by sodium dodecyl sulfate polyacrylamide gel electrophoresis (SDS-PAGE) and transferred onto a polyvinylidene difluoride (PVDF) membrane (Merck Millipore). Blots were probed with antibodies listed in Supplementary Material Table 1, incubated using Clarity ECL substrate (Bio-Rad) and imaged (Biospectrum imaging system, UVP). Densitometry was performed using Image Studio Lite software (LI-COR).

***NF-κB p65 staining and quantification***

Monolayers were fixed, permeabilised, blocked at room temperature with 2% bovine serum albumin in 0.1% Triton X (Sigma-Aldrich) for 1 h, incubated with antibodies listed in Supplementary Material Table 1 and imaged using a Leica SP5 inverted confocal microscope with a ×10, 0.40 NA objective. Example images are shown in Supplementary Figure 3. Nine images in a 3x3 grid were obtained from the centre of each target well and analysed using a custom script in MATLAB 2017b. Nuclei, stained with DRAQ5, were segmented from the background using intensity and area thresholding. The segmented nuclei were then used as a mask to quantify the pixel intensities of NF-κB p65 in the nuclei of the original images. Cytoplasmic NF-κB p65 was taken as all signal outside the nuclear mask. Translocation was obtained from the equation:

$$NF ̵\kappa B p65 Translocation= \frac{Mean Nuclear NF ̵\kappa B p65}{Mean Cytoplasmic NF ̵\kappa B p65}.$$

***THP-1 monocyte adhesion assay***

Suspension cultures of THP-1 cells were centrifuged at 210 x *g* for 5 minutes. The pellet was resuspended in RPMI 1640 at 5x10^5^ cells/mL. 1 μg/mL of Calcein-AM (Life Technologies) was added to the cell suspension, which was then incubated for 30 minutes in an incubator. The stained cells were spun down, resuspended in PAEC medium at 5x10^5^ cells/mL, and 1 mL of cell suspension were applied to each well containing a PAEC monolayer for 1 h. Non-adhered THP-1 monocytes were removed by gentle washing in PAEC medium three times and the wells were fixed with 4% paraformaldehyde for 15 minutes. Monolayers were imaged and THP-1 cells segmented as described for NF-κB immunostaining; the number of objects in the field was counted.

***Secretome analysis***

PAECs were seeded at the edge or centre of a 12-well plate and cultured with or without shear for 72 h, as above. The monolayers were washed with serum-free PAEC medium three times and cultured with or without shear for a further 24 h in serum-free medium. Conditioned medium was collected, centrifuged (10 minutes at 2000 x *g*, 4 °C) and stored at -80 °C.

Secretomes were concentrated using Amicon Ultra Centrifugal Filters (UFC900324) and protein concentrations were measured using the Pierce BCA Protein Assay Kit. 20 μg of protein extracts were denatured with urea and thiourea (final concentrations of 6 M and 2 M, respectively) and reduced with dithiothreitol (DTT, final concentration 10 mM) for 1 h at 37 °C. The samples were then alkylated by incubation with iodoacetamide (final concentration 50 mM) in the dark for 1 h at room temperature. Pre-chilled acetone (6x volume) was used to precipitate the samples overnight at -20 °C. Samples were centrifuged (25 minutes at 14000 x *g*, 4 °C) and the protein pellets were dried under vacuum, re-suspended in 0.1 M TEAB buffer (pH 8.2) containing trypsin/LysC (1:50 protease:protein) and digested overnight at 37 °C. Digestion was stopped by acidification of the samples with trifluoroacetic acid (TFA; final concentration 1%).

Peptide samples were purified using a 96-well C18 spin plate (Harvard Apparatus). The resin was activated using 200 µl methanol and centrifuged at 1000 x *g* for 1 minute. Wash steps included 200 µl of 80% acetonitrile (ACN), 0.1% TFA in H_2_O, and three equilibration steps using 200 µl of 1% ACN, 0.1% TFA in H_2_O with centrifugation (1000 x *g* for 1 minute) after each step. Samples were loaded onto the resin and centrifuged at 1500 x *g* for 1 minute; the flow through was reloaded onto the resin a second time and centrifugation repeated. The resin was then washed three times with 200µl 1% ACN, 0.1% TFA in H_2_O (centrifugation at 1500 x *g* for 1 minute). Finally, the samples were eluted with 170µl of 50% ACN, 0.1% TFA in H_2_O (centrifugation at 1500 x *g* for 1 minute) twice. The eluates were dried under vacuum and resuspended in 40 μL of 0.05% TFA in 2% ACN.

The peptide samples were separated on a nanoflow liquid chromatography (nano LC) system (Dionex UltiMate 3000 RSLC nano). Samples were injected onto a nano-trap column (Acclaim^®^ PepMap100 C18 Trap, 5mm x 300 μm, 5 μm, 100 Å), at a flow rate of 25 μL/min for 3 minutes, using 2% ACN, 0.1% formic acid (FA) in H_2_O. The following nano LC gradient was then run at 0.3 μL/min to separate the peptides: 0−10 min, 4-10% B; 10−75 min, 10-30% B; 75-80 min, 30−40% B; 80-85 min, 99% B, 90-120 min 4% B, where A=0.1% FA in H_2_O, B=80% ACN, 0.1% FA in H_2_O. The nano column (EASY-Spray PepMap RSLC C18, 2 μm, 100 Å, 75 µm x 50 cm) was connected to an EASY-Spray ion source (Thermo Scientific). Spectra were obtained with an Orbitrap mass analyser (Q Exactive HF, Thermo Fisher Scientific) using full MS mode (resolution of 60,000 at 200 m/z) over the mass-to-charge (m/z) range 350–1600. Data-dependent MS2 scanning was performed using the top 15 ions in each full MS scan (resolution of 15,000 at 200 m/z) with dynamic exclusion enabled.

Raw data files were searched against porcine Uniprot database (version 2020_01, 120,878 protein entries) combined with bovine database (UniProtKB/Swiss-Prot version 2020_01, 6,011 protein entries) using Thermo Scientific Proteome Discoverer software (version 2.4.0.305) and Mascot (version 2.6.0, Matrix Science). The mass tolerance was set at 10 ppm for precursor ions and 20 mmu for fragment ions. Trypsin was used as the enzyme with up to two missed cleavages being allowed. Carbamidomethylation of cysteine was chosen as a fixed modification; oxidation of methionine, deamidation of asparagine and N-terminal acetylation were chosen as variable modifications. Data were further processed using Scaffold (version 4.8.6) and the following filters were used: Peptide probability > 95%, Protein probability > 99% and with at least 2 peptides. Total ion current (TIC) was used for protein quantitation and normalized to total protein abundance. ANOVA was used to a p-value for each protein.

The mass spectrometry proteomics data have been deposited to the ProteomeXchange Consortium via the PRIDE [6] partner repository with the dataset identifier PXD022068 and 10.6019/PXD022068.

***FSTL1 measurement and application***

The concentration of FSTL1 in conditioned medium was detected by an enzyme-linked immunosorbent assay (ELISA, Abcam). Standards and samples were run in duplicate and optical density was measured at 450 nm using a plate reader (SpectraMax M5).

To determine the effect of exogenous FSTL1 on TNF-α-induced activation, 1μg/mL glycosylated FSTL1 (Life Technologies) was added 24 h before the TNF-α

To determine effects of FSTL1 on tracer transport, glycosylated FSTL1 (1 or 2 μg/mL) was added 24 h before the tracer studies. As a positive control, PAECs were treated with 80 μM Dynasore (Calbiochem) for 1 h before tracer studies.

***BMP4 and noggin measurement and application***

Human recombinant BMP4 and noggin protein were expressed in mouse myeloma cell lines (R&D systems). Cells were treated concurrently with BMP4, Noggin and/or or FSTL1. Tracer accumulation and western blot studies were conducted 24 h post-treatment as described above. The antibody was a mouse monoclonal anti-human BMP4 (R&D systems). BMP4 in conditioned medium was quantified using the Human BMP4 Quantikine ELISA kit (R&D systems).

***Measurement of LDL transport***

HAECs were seeded on Transwell inserts (Corning) pre-coated with 0.1% gelatin and cultured until confluent. The medium for the HAECs was replaced with reduced serum ECGM 24 h prior to permeability studies. Before the addition of LDL, filters were washed with serum free ECGM supplemented with 1% BSA and the compartments of the Transwell were allowed to reach equilibrium for 1 h. Finally, 0.5 mg protein/mL of LDL (Sigma-Aldrich)) was added to upper compartment. The medium in the bottom compartment was collected after 1 h. For wells treated with FSTL1, reduced serum ECGM and serum free ECGM were supplemented with 1% BSA that contained either 1 μg/mL glycosylated FSTL1 (Life Technologies) or 250 ng/mL non-glycosylated FSTL1 (Abcam).

Concentrations of LDL were detected using an ELISA kit (Cloud-Clone Corp). Standards and samples were run and measured as above.

***Statistical analysis***

Statistical significance of results was assessed using Student’s t-test or a one-way ANOVA followed by Tukey multiple-comparison *post hoc* test if ANOVA revealed significance between means. P < 0.05 was used as the criterion for significance and *, **, and *** denote P < 0.05, P < 0.01, P < 0.001, respectively. n is the number of independent experiments.

***References***

1. Bogle RG, Baydoun AR, Pearson JD, Mann GE. Regulation of l-arginine transport and nitric oxide release in superfused porcine aortic endothelial cells. J Physiol. 1996;490:229–241.

2. Ghim M, Pang KT, Arshad M, Wang X, Weinberg PD. A novel method for segmenting growth of cells in sheared endothelial culture reveals the secretion of an anti-inflammatory mediator. J Biol Eng. 2018;12:15.

3. Pang KT, Ghim M, Arshad M, Wang X, Weinberg PD. Segmenting growth of endothelial cells in 6-well plates on an orbital shaker for mechanobiological studies.” J Vis Exp. 2021; 172: e61817.

4. Ghim M, Alpresa P, Yang S, Braakman ST, Gray SG, Sherwin SJ, van Reeuwijk M, Weinberg PD. Visualisation of three pathways for macromolecule transport across cultured endothelium and their modification by flow. Am J Physiol Heart Circ Physiol. 2017;313:H959-H973.

5. Dubrovskyi O, Birukova AA, Birukov KG. Measurement of local permeability at subcellular level in cell models of agonist- and ventilator-induced lung injury. Lab Invest. 2013;93:254-263.

6. Perez-Riverol Y, Csordas A, Bai J, Bernal-Llinares M, Hewapathirana S, Kundu DJ, Inuganti A, Griss J, Mayer G, Eisenacher M, Pérez E, Uszkoreit J, Pfeuffer J, Sachsenberg T, Yilmaz S, Tiwary S, Cox J, Audain E, Walzer M, Jarnuczak AF, Ternent T, Brazma A, Vizcaíno JA. The PRIDE database and related tools and resources in 2019: improving support for quantification data. Nucleic Acids Res 2019;47(D1):D442-D450.

**Supplementary Material Table 1: Antibodies**

| **Target** | **Vendor** | **Catalog** | **Dilution** | **URL** |
| --- | --- | --- | --- | --- |
| Rabbit anti human NF-κB p65 | Santa Cruz Biotechnology | sc-372 | 1:200 | https://www.scbt.com/p/nfkappab-p65-antibody-c-20 |
| AlexaFluor 488-labelled goat anti-rabbit IgG | Life Technologies | A-11034 | 1:300 | https://www.thermofisher.com/antibody/product/Goat-anti-Rabbit-IgG-H-L-Highly-Cross-Adsorbed-Secondary-Antibody-Polyclonal/A-11034 |
| Rabbit anti-human VCAM-1 | Santa Cruz Biotechnology | sc-8304 | 1:2000 | https://www.scbt.com/p/vcam-1-antibody-h-276 |
| Rabbit anti-human ICAM-1 | Santa Cruz Biotechnology | sc-7891 | 1:2000 | https://www.scbt.com/p/icam-1-antibody-h-108 |
| Rabbit anti-human p-Iκbα | Cell Signaling Technology | 2859s | 1:1000 | https://www.cellsignal.com/products/primary-antibodies/phospho-ikba-ser32-14d4-rabbit-mab/2859?Ntk=Products&Ntt=2859 |
| Rabbit anti-human IκBα | Santa Cruz Biotechnology | sc-371 | 1:2000 | https://www.scbt.com/p/ikappab-alpha-antibody-c-21 |
| Mouse anti-human BMP4 | R&D Systems | MAB757 | 1:2000 | https://www.rndsystems.com/products/human-bmp-4-antibody-66119_mab757 |
| Goat anti-human GAPDH | Santa Cruz Biotechnology | sc-25778 | 1:2000 | https://www.scbt.com/p/gapdh-antibody-fl-335 |
| Mouse anti-goat horseradish peroxidase-conjugated secondary | Santa Cruz Biotechnology | Sc-2354 | 1:5000 | https://www.scbt.com/p/mouse-anti-goat-igg-hrp |

Supplementary Figure I: Computer aided design (CAD) drawings for the mask moulds for A) edge and C) centre, and for the masks for B) edge and D) centre***.***

Supplementary Figure 2: Images of DAPI stained PAEC nuclei. PAECs were cultured at **A**) the edge or **B**) the centre of a 12-well plate for five days under static conditions. Bar = 1 mm and 100 μm in left and right images respectively.

Supplementary Figure 3 (next page): Representative images showing immunostaining of NF-κB p65 (green) and nuclear staining with DRAQ5 (red) in PAECs that were untreated or treated with TNF-α. Following TNF-α treatment, some cells were additionally treated conditioned medium (CM) from cells grown under static conditions or cells grown at the centre or edge of swirled wells. Bar = 500 μm

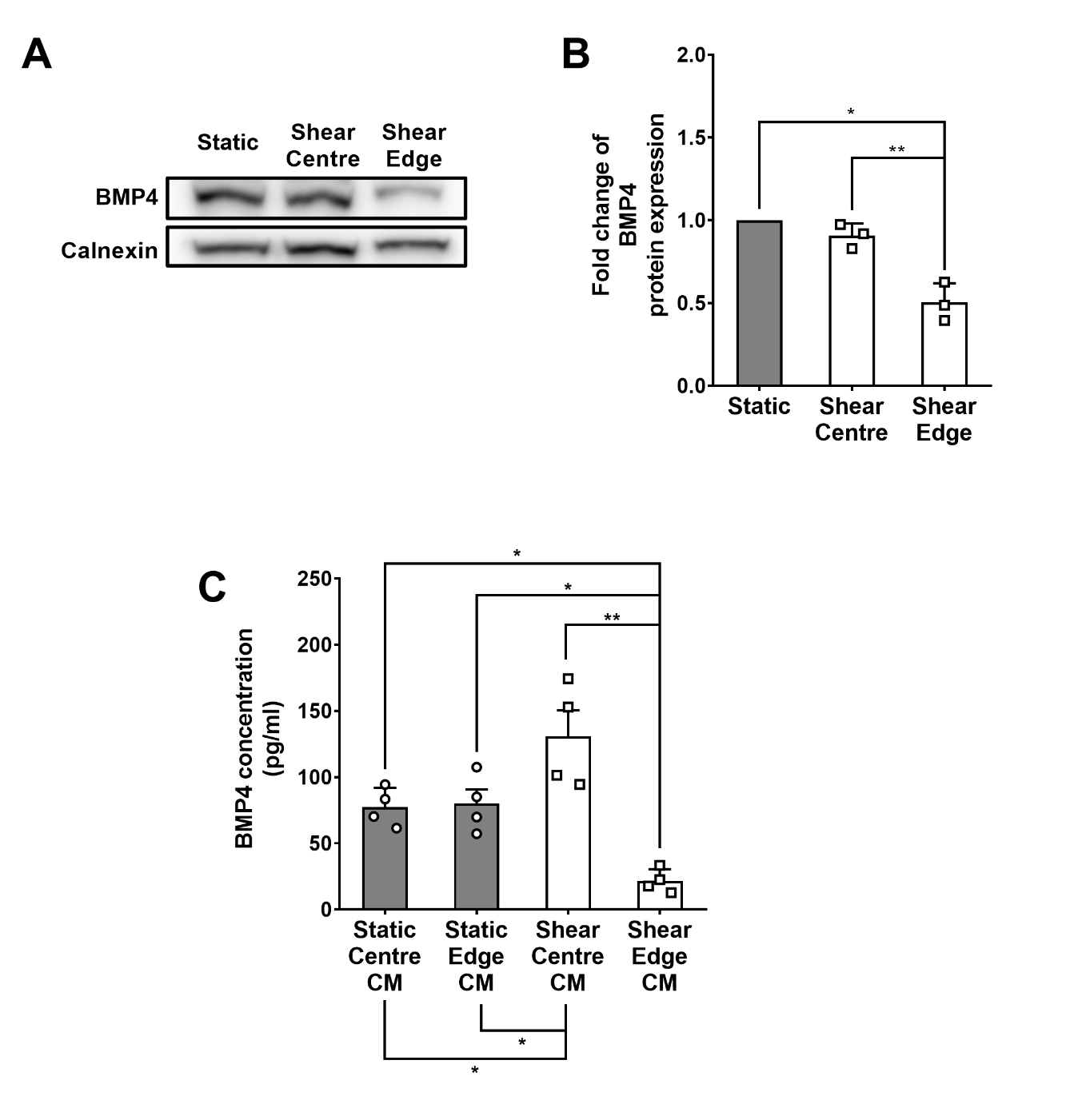


Supplementary Figure 4: **A**) Western blot of BMP4 and calnexin in HAECs cultured under static conditions or swirled at the centre or edge of the well. **B**) Quantification of western blots to show expression of BMP4 in HAECs cultured under static conditions or swirled at the centre or edge of the well. **C**) Quantification by ELISA of the concentration of BMP4 in medium conditioned by HAECs cultured at the centre or edge of static or swirled wells.
